# Perceptual load vs. dilution: the roles of attentional focus, stimulus category, and target predictability

**DOI:** 10.3389/fpsyg.2013.00327

**Published:** 2013-06-07

**Authors:** Zhe Chen, Kyle R. Cave

**Affiliations:** ^1^Department of Psychology, University of CanterburyChristchurch, New Zealand; ^2^Department of Psychology, University of MassachusettsAmherst, MA, USA

**Keywords:** selective attention, distractor interference, perceptual load, dilution, attentional focus

## Abstract

Many studies have shown that increasing the number of neutral stimuli in a display decreases distractor interference. This result has been interpreted within two different frameworks; a perceptual load account, based on a reduction in spare resources, and a dilution account, based on a degradation in distractor representation and/or an increase in crosstalk between the distractor and the neutral stimuli that contain visually similar features. In four experiments, we systematically manipulated the extent of attentional focus, stimulus category, and preknowledge of the target to examine how these factors would interact with the display set size to influence the degree of distractor processing. Display set size did not affect the degree of distractor processing in all situations. Increasing the number of neutral items decreased distractor processing only when a task induced a broad attentional focus that included the neutral stimuli, when the neutral stimuli were in the same category as the target and distractor, and when the preknowledge of the target was insufficient to guide attention to the target efficiently. These results suggest that the effect of neutral stimuli on the degree of distractor processing is more complex than previously assumed. They provide new insight into the competitive interactions between bottom-up and top-down processes that govern the efficiency of visual selective attention.

## Introduction

When a target is presented with distractors in a search array, the distractors are often processed to some extent along with the target, resulting in increased response latencies when the target and distractors indicate different responses compared with when they indicate the same response (e.g., Eriksen and Hoffman, 1973; Eriksen and Eriksen, 1974; Miller, 1987). This response congruency effect has been observed in a variety of paradigms (e.g., Eriksen and St. James, 1986; Kramer and Jacobson, 1991; Chen and Cave, 2006). Even when a search array appears to facilitate target selection via optimal attentional focusing, evidence of distractor processing has still been found (e.g., Gatti and Egeth, 1978; Miller, 1991), showing that attentional selection is often inefficient. Understanding the mechanisms that modulate the degree of distractor processing is important, because it helps to shed light on the locus of attentional selection and how bottom-up and top-down processes interact in visual information processing. This study focuses on the factors that affect distractor processing in visual search.

Although the response congruency effect is frequently observed in selective attention tasks, it does not always appear. Lavie and Tsal (1994) and Lavie (1995) noted that the magnitude of the effect, which indicates the degree of distractor processing, is closely linked to the perceptual load required of a task, with a larger effect associated with a low perceptual load task and a smaller effect with a high perceptual load task. For example, in one experiment (Lavie, 1995, Experiment 1), Lavie varied the target-distractor response congruency (congruent, neutral, and incongruent) and the perceptual load involved in selecting the target (low vs. high). Perceptual load was manipulated via adjusting the number of elements in the display. In the low load condition, the target was shown with a single distractor. In the high load condition, it was shown with several neutral stimuli in addition to the distractor. A larger response congruency effect was found in the low compared with the high perceptual load condition. Based on this and other similar results, [Lavie (1995); see also Lavie and Tsal (1994)] proposed a perceptual load theory, in which perception is an automatic process with a limited pool of resources. To the extent there is spare capacity beyond what is used in processing the target, perception proceeds involuntarily until all the resources are used up. When a task involves a low perceptual load, distractor processing occurs because of the spillover resources. When a task involves a high perceptual load, distractor processing is either reduced or eliminated due to the unavailability of spare resources. Thus, the degree of distractor processing depends on the amount of leftover resources, which, in turn, is determined by the perceptual load of a task. Since its proposal, evidence in support of the perceptual load theory has been reported in many studies (see Lavie, 2005, for a review).

However, despite this supporting evidence, there is also a growing number of studies that have shown results inconsistent with the perceptual load theory. Whereas the typical perceptual load effect, i.e., a large response congruency effect with low perceptual load, was observed when the low and high perceptual load trials were presented in separate blocks (e.g., Lavie, 1995; Lavie and Cox, 1997; Lavie and Fox, 2000), the effect was reduced or even eliminated when the two types of trials were intermixed within the same block (Murray and Jones, 2002; Theeuwes et al., 2004). The perceptual load effect was also eliminated, and sometimes even reversed, when the location of the target was precued (Paquet and Craig, 1997; Johnson et al., 2002), when the target and distractor were placed in separate objects or perceptual groups (Baylis and Driver, 1992; Tsal and Benoni, 2010; Cosman and Vecera, 2012; Yeh and Lin, 2013), and when the relevant and irrelevant information belonged to the same object (Chen, 2003). Other factors such as the number of locations at which a distractor or a target could appear (Marciano and Yeshurun, 2011; Wilson et al., 2011) and the relative salience of a target and distractor (Eltiti et al., 2005) also influenced the degree of distractor processing in ways inconsistent with the perceptual load theory. Together, these results challenge the perceptual load theory. They suggest that perceptual load, instead of being a determinant in distractor processing as proposed by the perceptual load theory, is one of a number of factors that contribute to the degree of distractor processing.

Recently, several researchers (Benoni and Tsal, 2010; Tsal and Benoni, 2010; Wilson et al., 2011) proposed an alternative account of distractor processing. Tsal and Benoni (2010) noted that evidence supporting the perceptual load theory came largely from experiments that manipulated perceptual load via display set size. Because an increase in display set size entails an increase in the number of neutral stimuli, and previous research on Stroop interference has shown that increasing irrelevant stimuli in a Stroop display dilutes Stroop interference (Kahneman and Chajczyk, 1983; Brown et al., 1995), this raises the question whether the reduction in distractor processing in a high perceptual load task is caused by the dilution of distractor interference rather than by the unavailability of perceptual resources. To test their hypothesis, Tsal and Benoni measured distractor interference in three types of displays: the typical low and high perceptual load displays that differed in the number of neutral stimuli, and a new dilution display that had the same number of neutral stimuli as that in the high load display but differed from the high load display in that the target and the neutral stimuli were perceptually segregated by color or spatial location. This segregation made it easy for the neutral stimuli to be ignored, so that the dilution display was low in perceptual load but high in display set size. Contrary to the prediction of the perceptual load theory, no response congruency effect was found in the dilution condition.

Based on this and similar results from other experiments, Tsal and Benoni (2010) proposed a dilution account of distractor processing. According to this account, an incongruent distractor causes interference when its representation is sufficiently strong to enter lexical memory and activate the target-opposite response category. When neutral stimuli, regardless of their task relevancy, are present in a display, their features compete with those of the incongruent distractor, degrading the quality of its representation. When the degraded representation of the distractor is not strong enough to enter lexical memory, there can be little distractor interference. In other words, it is the dilution of distractor interference, not the unavailability of spare perceptual resources that eliminates distractor interference in displays with a large set size.

Wilson et al. (2011) proposed a slightly different dilution account to interpret the display set size effect in distractor processing. They proposed a two-stage model, following from Neisser (1967) and Hoffman (1979): there is an initial parallel processing stage, during which the location most likely to contain the target is selected, and a serial processing 2nd stage, during which the selected item is further processed. Because only one item is processed at a time in the 2nd stage, all the other stimuli in the search array are irrelevant in that stage, and this is so regardless of whether a specific stimulus is task relevant or irrelevant in the 1st stage. Dilution occurs during the 2nd stage if there are sufficient spare resources to process the irrelevant stimuli. Increasing the number of neutral stimuli reduces distractor processing, either because of decreased resources to each stimulus or because of increased crosstalk between the distractor and the neutral stimuli. Thus, like Tsal and Benoni (2010), Wilson et al. attribute the display set size effect to the presence of neutral stimuli, which dilute distractor interference regardless of their task relevancy. They manipulated both the display set size and the number of locations at which a target could appear so that the neutral stimuli were relevant on some trials and not on the other trials. The response congruency effect decreased with increasing display set size, and as they predicted, the reduction was comparable regardless of the relevancy of the neutral stimuli to the task. These results are consistent with the notion that the mere presence of neutral stimuli dilutes distractor interference. Experiment 1 below provides an illustration of some of the main aspects of Wilson et al.'s experiments, and replicates their results.

Wilson et al. (2011) found that the dilution effect was comparable regardless of the cued target locations, but previous research generally shows that the attentional focus modulates the degree of distractor processing. The idea of attentional focus was captured in Eriksen and St. James' (1986) “zoom lens model,” and it was described by Cave et al. (2010) as “attentional zoom,” and by Wilson et al. as “attentional breadth.” Using a spatial cuing paradigm, Yantis and Johnston (1990) reported that presenting a 100% valid cue before the onset of a target could minimize distractor interference in a search display. Paquet and Lortie (1990) also reported that precuing the target location decreased distractor interference when the target and distractors belonged to the same category. Similar results were shown by LaBerge and his colleagues (1991), who demonstrated that narrowing attention focus so that the distractor appeared outside it decreased distractor interference, and by Eriksen and St. James (1986), who found reduced distractor interference when the number of precued locations decreased. In both cases, an incongruent distractor caused less interference when a task induced a relatively small attentional focus that excluded the distractor. Thus, all else being equal, a larger response congruency effect is more likely to be found when an incongruent distractor is inside rather than outside an observer's attentional focus.

Attentional focus has also been shown to mediate the effect of perceptual load on distractor processing. For example, when a 100% valid precue was used to indicate the location of the target, the perceptual load effect was eliminated (Johnson et al., 2002). The perceptual load effect was also reduced or eliminated when participants were prevented from varying the extent of attentional focus between the low and high load trials, either by intermixing the two types of trials within the same block (Murray and Jones, 2002; Theeuwes et al., 2004) or by designing stimuli so that the relevant and irrelevant information pertained to the same object (Chen, 2003). Furthermore, intertrial analyses showed that distractor interference on a high perceptual load trial was more likely to occur when it was preceded by a low perceptual load trial rather than by a high perceptual load trial (Theeuwes et al., 2004; Biggs and Gibson, 2010). As low perceptual load is more likely to induce a relatively broad attentional focus compared with high perceptual load, the observed intertrial contingency, together with the finding that intermixing trials of different perceptual loads within the same block could reduce or eliminate the perceptual load effect, suggests that different response strategies, with variations in attentional focus, may have played a role in the perceptual load effect found in many previous studies.

The results of recent research underscore the importance of understanding the roles of neutral stimuli and attentional focus, and how they interact to influence distractor processing in visual selection. In Wilson et al. (2011), the magnitude of dilution was comparable regardless of the number of cued target locations. Because the extent of attentional focus should correlate with the cue set size, this result indicates that the extent of attention focus did not affect dilution effects. In other words, in Wilson et al.'s study, whether a stimulus was located inside or outside an observer's attentional focus did not influence the degree of processing of that stimulus. As we discussed in the previous section, this finding conflicts with previous research, which shows that a stimulus receives more processing when it is inside rather than outside one's attentional focus (e.g., Eriksen and St. James, 1986; Yantis and Johnston, 1990; LaBerge et al., 1991).

In Wilson et al. (2011)'s study, the appearance of the target display was marked by onset transients and the total number of stimuli in the target display varied in accordance with the display set size. Abrupt visual onsets attract attention under most circumstances (Yantis and Jonides, 1984, 1990). Consequently, the use of onset transients in Wilson et al.'s experiments could undermine the spatial distribution of attention induced by the cue, resulting in a larger attentional focus when the display set size was large rather than when it was small. This could lead to the comparable dilution effects in both the small and large cue set size conditions in Wilson et al.'s study.

The 4 experiments reported in this study investigated the factors that influence dilution effects. Specifically, we focused on three issues: the role of attentional focus in modulating the effect of display set size on distractor processing, the locus of dilution, and the role of target knowledge in dilution effects. In Experiment 1, we deliberately co-varied display set size with the extent of attentional focus by using luminance increment to signal the appearance of the target display. Our goal was to replicate the findings of Wilson et al. (2011), and we did. The magnitude of the dilution effect was similar regardless of whether 2 or 6 target locations were cued. Experiment 2 used luminance decrement instead of luminance increment so that the stimulus change lowered the contrast rather than raising it, and thus the attentional focus induced by the cue would not be affected very much by the appearance of the target display. A dilution effect was found when the extent of attentional focus was large, but not when it was small. In Experiment 3, we explored the locus of dilution by varying the number of inverted letters in the two display set size conditions. No dilution effects were found, suggesting that dilution occurred beyond a feature level. Finally, in Experiment 4, we tested the effect of preknowledge of the target by making its color predictable for one group of participants but unpredictable for the other group. A dilution effect was found for the latter group, but not for the former one.

## Experiment 1

**Figure 1:**
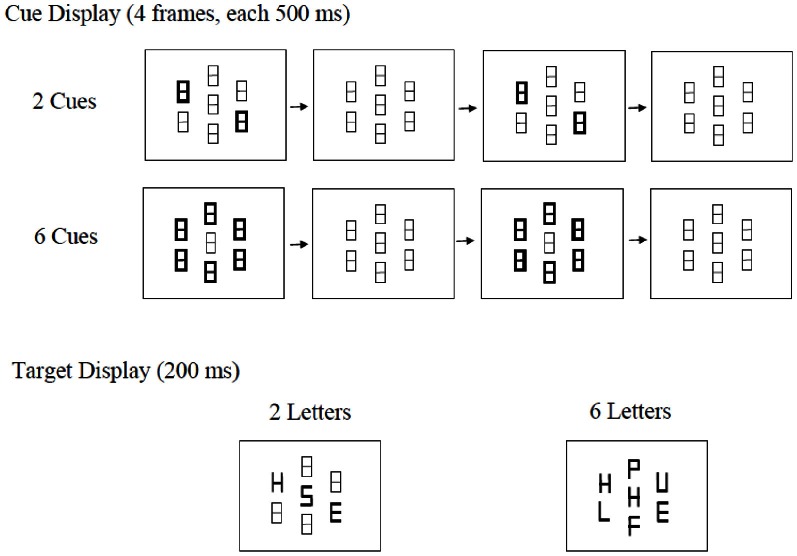
**Examples of cue displays and target displays from Experiment 1.** The cue display consisted of 4 frames. The locations of the target, which was either an H or an S, were indicated by 2 or 6 figure-8 place-holders increasing in luminance. The target display consisted of 2 letters or 6 letters, excluding the critical distractor, which always appeared at the center. The 2-letter display is an example of an incongruent trial. The 6-letter display is an example of a congruent trial. Note that the appearance of the target display is signaled by luminance increment.

Experiment 1 was modeled after Wilson et al. (2011) to replicate their results with our modified experimental paradigm, which differed from Wilson et al.'s in that the number of stimuli in the target display was held constant via the use of non-letter place-holders. As in Wilson et al., we manipulated cue set size (CueSize) and display set size (DisplaySize) independently (see Figure 1). CueSize refers to the number of possible locations at which a target could appear (2 or 6), and DisplaySize refers to the number of letters in the search array (2 or 6, excluding the critical distractor). Luminance increment was used to signal the appearance of the target display. There was always one target letter present in each display, either an H or an S, and the task was to determine as quickly and as accurately as possible which of the two targets was present. Based on Wilson et al.'s results, we expected our participants to show a dilution effect of similar magnitude in both the 2-cue and 6-cue conditions.

### Method

#### Participants

Nineteen undergraduate students from the University of Canterbury volunteered to participate in the experiment. Each was paid NZ$10. All reported to have normal or corrected-to-normal vision.

#### Apparatus and stimuli

Stimulus displays were shown on a PC with a 16-inch monitor. The participants were tested individually in a dimly lit room. The viewing distance was approximately 60 cm. E-prime 2.0 (Schneider et al., 2002) was used to display stimuli and to record responses.

All stimuli were presented against a black background. Each trial consisted of three displays: the fixation, the cue, and the target display. The fixation display consisted of 7 identical gray (RGB = 60, 60, 60) figure-8 stimuli that also served as place-holders in subsequent displays. Each place-holder subtended 0.86° of visual angle in height and 0.57° in width. While six of them were placed at equal distance along the perimeter of an imaginary circle centered on fixation with a radius of 2.48°, the 7th one was always at fixation. The cue display consisted of four frames. Frames 2 and 4 were identical to the fixation display. Frames 1 and 3 differed in that either a pair of place-holders in opposite locations (in the 2-cue condition) or all six place-holders along the imaginary perimeter (in the 6-cue condition) became white (RGB = 255, 255, 255) instead of remaining to be gray. As there was no blank screen between the fixation and the cue display or between any two frames in the cue display, the perception of the cue was that of 2 or 6 place-holders flashing twice.

We are using the DisplaySize label to be consistent with Wilson et al. (2011), but when the DisplaySize was 2 in this experiment, there were 4 figure-8 place-holders added to the display, so that the total number of stimuli with the distractor and the place-holders was always 7. Following Yantis and Jonides (1984), the letters, which were white in color (RGB = 255, 255, 255), were constructed by increasing the luminance of the appropriate line segments of the figure-8 stimuli and deleting the unneeded segments. Thus, the letters were created via luminance increment rather than the onset transients used by Wilson et al. The stimulus at fixation was always the critical distractor. It was white, and was equally likely to be an H or an S. In the 6-letter condition, the search array consisted of a target letter (H or S) and 5 neutral letters (P, E, F, L, and U). In the 2-letter condition, the search array consisted of a target (again an H or an S), a neutral letter selected randomly and with equal probability from the set of neutral letters mentioned above, and 4 place-holders identical to those in the fixation display. On half of the trials (the congruent condition), the target and distractor were identical. On the rest of the trials (the incongruent condition), they were different letters associated with different responses.

#### Design and procedure

The experiment used a 2 × 2 × 2 within-participants design. The principal manipulations were CueSize (2-cue vs. 6-cue), DisplaySize (2-letter vs. 6-letter), and target-distractor Congruency (congruent vs. incongruent). The three factors were varied independently. All types of trials were presented randomly within a block.

Each trial started with the presentation of the fixation display. After 500 ms, either 2 or 6 place-holders along the perimeter of the imaginary circle would flash twice, with each flash lasting for 500 ms, with a 500 ms interval after each flash. At the end of the 2nd interval (i.e., the 4th frame of the cue display), the central place-holder would turn into a letter, as would either 2 or 6 of the other place-holders, depending on the DisplaySize condition. The screen went black after 200 ms. The task was to respond, as quickly and as accurately as possible, whether the target was an H or an S. The participants were instructed to maintain fixation at the central place-holder throughout the duration of a trial, and to use the index and middle fingers of their right hand to press one of the two designated keys on a response box (the 4th key if the target letter was an “H,” and the 5th key if it was an “S”). They were explicitly informed that the target would only appear at one of the cued locations and that the center letter was always a distractor that they should try to ignore. The entire experiment consisted of 2 blocks of 16 practice trials, followed by 5 blocks of 96 experimental trials with short breaks after each block. It took about 35 min to complete the experiment.

### Results

**Figure 2:**
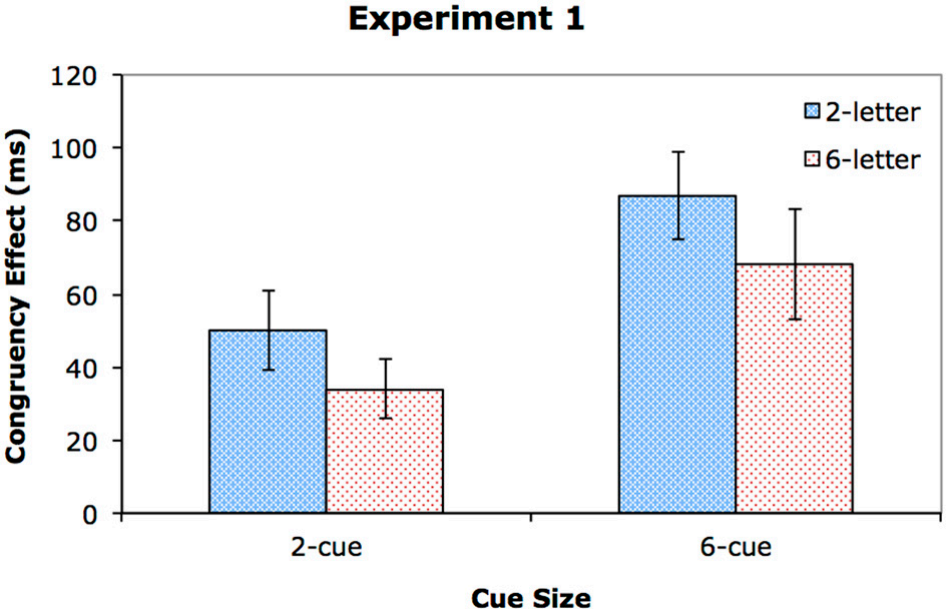
**The congruency effect (incongruent RT–congruent RT) across the different conditions of Experiment 1.** Error bars show the standard error of the mean.

Table 1 shows the mean reaction times (RTs) for correct responses and the error rates, and the graph in Figure 2 shows the congruency effect across conditions^1^. One participant's data were not included due to high error rates (greater than 40% in one condition). A 2 × 2 × 2 repeated measures analysis of variance (ANOVA) was conducted on RTs. (See Table 2 for details of the results). All the main effects were significant. The participants were faster in the 2-cue condition (655 ms) than in the 6-cue condition (759 ms), *p* < 0.001. They were also faster when the display consisted of two letters (654 ms) instead of six letters (760 ms), *p* < 0.001, and when the target and distractors were congruent (677 ms) rather than incongruent (737 ms), *p* < 0.001. CueSize interacted with DisplaySize, *p* < 0.001, suggesting that RT increased more dramatically from the 2-letter condition to the 6-letter condition on the 6-cue trials (an increase of 162 ms) than on the 2-cue trials (an increase of 51 ms). CueSize also interacted with Congruency, *p* < 0.02. The congruency effect was larger in the 6-cue condition (78 ms) than in the 2-cue condition (42 ms), indicating a positive relationship between the number of target locations and the degree of distractor processing. Furthermore, a dilution effect was found, as evidenced by the significant interaction between DisplaySize and Congruency, *p* < 0.02, suggesting a larger congruency effect in the 2-letter condition (69 ms) than in the 6-letter condition (51 ms). Finally, there was no significant three-way interaction of CueSize, DisplaySize, and Congurency. The last result indicated that the magnitude of the dilution effect was independent of the cue set size, as can be seen in Figure 2.

**Table 1:** **Experiment 1: mean reaction times and error rates as a function of cue set size, display set size, and target-distractor congruency**.

**Display set size**	**Cue set size**			
	**2-cue**		**6-cue**	
	***C***	***I***	***C***	***I***
**REACTION TIMES (ms)**				
2-letter	604 (22)	654 (23)	635 (22)	722 (26)
6-letter	663 (34)	697 (31)	806 (27)	874 (35)
**ERROR RATES (% INCORRECT)**				
2-letter	2.2 (0.6)	4.7 (0.9)	3.7 (0.8)	7.0 (1.5)
6-letter	6.7 (1.1)	6.0 (1.1)	13.3 (1.7)	12.4 (1.9)

Standard errors are in the parentheses. C, Congruent; I, Incongruent.

**Table 2:** **Results of statistical analyses of the reaction times in Experiments 1, 2, and 3**.

	**Reaction times**								
	**Experiment 1**			**Experiment 2**			**Experiment 3**		
	***F*_(1, 17)_**	***p***	**η^2^_*p*_**	***F*_(1, 19)_**	***p***	**η^2^_*p*_**	***F*_(1,15)_**	***p***	**η^2^_*p*_**
Cue	34.82^***^	0.001	0.67	124.85^***^	0.001	0.87	174.00^***^	0.001	0.92
Display	73.48^***^	0.001	0.81	90.02^***^	0.001	0.83	77.63^***^	0.001	0.84
Cong	64.77^***^	0.001	0.79	58.76^***^	0.001	0.76	48.93^***^	0.001	0.77
Cue^*^Display	25.36^***^	0.001	0.60	60.74^***^	0.001	0.76	54.58^***^	0.001	0.78
Cue^*^Cong	6.87^*^	0.02	0.29	16.14^***^	0.001	0.46	24.70^***^	0.001	0.62
Display^*^Cong	6.94^*^	0.02	0.29	3.00	0.10	0.14	0.02	0.89	0.01
Cue^*^Display^*^Cong	0.03	0.86	0.01	9.44^**^	0.01	0.33	0.06	0.81	0.01

Cue, CueSize; Display, DisplaySize; Cong, Congruency.

* p < 0.05;

** p < 0.01;

*** p < 0.001.

A similar ANOVA was conducted on the error rates. (See Table 3 for details of the results). Consistent with the RT results, error rates were lower in the 2-cue condition (4.9%) than in the 6-cue condition (9.1%), *p* < 0.001, and on the 2-letter trials (4.4%) than on the 6-letter trials (9.6%), *p* < 0.001. CueSize interacted with DisplaySize, *p* < 0.02, suggesting a larger increase in error rate from the 2-letter to 6-letter condition on the 6-cue trials (an increase of 7.5%) compared with the 2-cue trials (an increase of 2.9%). Finally, there was a significant interaction between DisplaySize and Congruency, *p* < 0.02. Whereas a significant congruency effect was found on the 2-letter trials (2.9% error rate), a similar effect was not found on the 6-letter trials (−0.8% error rate). No other effects reached significance. There was no indication of any speed-accuracy tradeoff.

**Table 3:** **Results of statistical analyses of the error rates in Experiments 1, 2, and 3**.

	**Error rates**								
	**Experiment 1**			**Experiment 2**			**Experiment 3**		
	***F*_(1, 17)_**	***p***	**η^2^_*p*_**	***F*_(1, 19)_**	***p***	**η^2^_*p*_**	***F*_(1, 15)_**	***p***	**η^2^_*p*_**
Cue	27.09^***^	0.001	0.61	52.68^***^	0.001	0.73	57.21^***^	0.001	0.79
Display	36.96^***^	0.001	0.68	69.07^***^	0.001	0.78	28.30^***^	0.001	0.65
Cong	1.85	0.19	0.10	7.77^*^	0.02	0.29	6.85^*^	0.02	0.31
Cue^*^Display	7.04^*^	0.02	0.29	79.22^***^	0.001	0.81	16.85^***^	0.001	0.53
Cue^*^Cong	0.07	0.80	0.01	2.24	0.15	0.11	2.41	0.14	0.14
Display^*^Cong	7.99^*^	0.01	0.32	0.10	0.75	0.01	0.01	0.95	0.01
Cue^*^Display^*^Cong	0.18	0.68	0.01	0.12	0.73	0.01	0.04	0.84	0.01

Cue, CueSize; Display, DisplaySize; Cong, Congruency.

* p < 0.05;

*** p < 0.001.

### Discussion

The results of Experiment 1 were remarkably similar to those of Wilson et al. (2011). In both cases, the congruency effect was substantially larger in the 6-cue condition than in the 2-cue condition. As they pointed out, this result is inconsistent with the perceptual load theory, which predicts a decrease in distractor interference with increasing cue set size, because perceptual load would increase with the number of locations at which a target could appear. Indeed, if RT is a valid indicator of perceptual load, then the longer RT in the 6-cue than the 2-cue condition provides evidence for the higher perceptual load in the former than in the latter. The fact that the perceptual load effect was reversed across the cue conditions is incompatible with the perceptual load theory.

The larger congruency effect in the 6-cue condition was likely caused by the increased RT in that condition compared with the 2-cue condition. As the cue in the 6-cue condition would induce a broader attentional focus than the cue in the 2-cue condition, more irrelevant letters would be within the attentional focus in the former condition, resulting in longer response latencies to the target. Previous research has shown a positive link between the processing time of a target and the magnitude of the congruency effect, and it has been proposed that an increase in the processing time of a target increases the window of opportunity for distractor intrusion, resulting in increased distractor processing (Lavie and De Fockert, 2003; Tsal and Benoni, 2010; Wilson et al., 2011). We agree with this view, and attribute the differential congruency effects in the two cue size conditions to the longer response latencies in the 6-cue condition relative to the 2-cue condition.

As in Wilson et al. (2011), we found that the congruency effect was more diluted when there were more letters in the display, and more importantly, the degree of dilution was comparable in both the 2-cue and 6-cue conditions. However, as we discussed before, the luminance increment that was used to signal the appearance of the target display in the present experiment, which is similar to the onset transient used in Wilson et al.'s (2011) experiments, could change the extent of attentional focus, raising doubts about the ability to measure the effects of perceptual load and dilution. In Experiment 2, we addressed this issue by using luminance decrement instead of luminance increment to minimize the effect of stimulus appearance on the extent of attentional focus induced by the cue.

## Experiment 2

**Figure 3:**
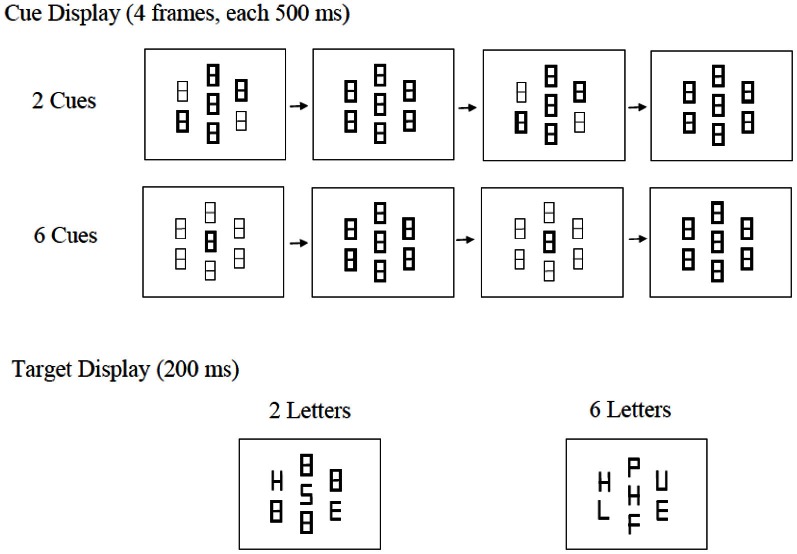
**Examples of cue displays and target displays from Experiment 2.** Note that the appearance of the target display is signaled by luminance decrement.

In Experiment 2, we replaced luminance increment with luminance decrement so that the target locations in the cue display and the appearance of the letters in the target display were both signaled via luminance decrease instead of luminance increase (see Figure 3). Because luminance decrement is less likely to capture attention than luminance increment (Yantis and Jonides, 1984), the appearance of the target display should be less likely to affect the extent of attentional focus induced by the cue, allowing the attentional focus to be determined more by the manipulation in CueSize.

As our selective review of the literature in the previous section indicates (e.g., Paquet and Lortie, 1990; Paquet and Craig, 1997; Johnson et al., 2002), there is reason to believe that the effect of neutral stimuli on distractor processing could be strongly affected in this paradigm by their locations relative to the attentional focus. As stimuli are likely to be processed at a greater extent when they are inside rather than outside one's attentional focus, we predicted a larger dilution effect in the 6-cue condition compared with the 2-cue condition, for more neutral letters should fall inside the participants' attentional focus in the former than in the latter.

### Method

The method of Experiment 2 was the same as that in Experiment 1 except for the following differences. First, the place-holders in the fixation display were white instead of gray. Second, target locations were indicated by luminance decrement instead of luminance increment in the cue display. Frames 2 and 4 were identical to the fixation display, i.e., all the place-holders were white. This ensured that compared with the participants in Experiment 1, those in Experiment 2 were less likely to expand their attentional focus upon the onset of the target display in the 2-cue condition, for the appearance of the target display was signaled by luminance decrement instead of luminance increment. Frames 1 and 3 differed from the fixation display in that the 2 or 6 place-holders in the cued locations were gray. Thus, the perception of the cue was that of 2 or 6 place-holders dimming twice. Third, in the target display, all the stimuli were white regardless of whether they were letters or place-holders. These design features ensured that there was minimal difference in luminance from the last frame of the cue to the target display, or between the target displays in the 2-letter and 6-letter conditions. Twenty new participants took part in the experiment.

### Results

**Figure 4:**
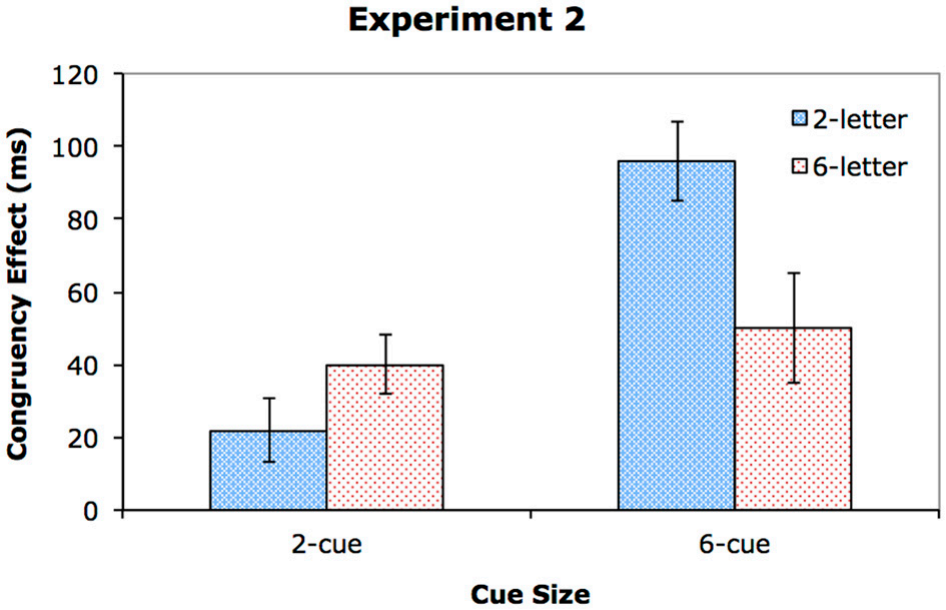
**The congruency effect for Experiment 2**.

Table 4 shows the mean RTs and error rates, and Figure 4 shows the effects of congruency. Two repeated measures ANOVAs were conducted, one on the RT data (see Table 2), and the other on the error rates (see Table 3). As in Experiment 1, all the three main effects were significant. The participants were faster and more accurate in the 2-cue condition (613 ms with 5.7% error rate) than in the 6-cue condition (757 ms with 11.5% error rate), *p* < 0.001, for both RT and error rates. They were also faster and more accurate in the 2-letter condition (653 ms with 6.3% error rate) than in the 6-letter condition (717 ms with 10.9% error rate), *p* < 0.001 in both cases. In addition, performance was better on congruent trials (659 ms with 7.2% error rate) than on incongruent trials (711 ms with 10.1% error rate), *p* < 0.001 for RT; and *p* < 0.02 for error rates. CueSize interacted with DisplaySize, both in RT, *p* < 0.001, and in error rates, *p* < 0.001, suggesting that an increase in display set size impaired performance more when the target could appear at 1 of 6 locations (an increase of 115 ms and 8.8% error rate) rather than at 1 of 2 locations (an increase of 14 ms and 0.5% error rate). In RT, the magnitude of the congruency effect was again affected by CueSize, *p* < 0.001. The congruency effect was larger in the 6-cue condition (73 ms) than in the 2-cue condition (31 ms). Finally, there was a significant three-way interaction in RT, *p* < 0.01, which is illustrated in Figure 4. No other effects reached significance.

**Table 4:** **Experiment 2: mean reaction times and error rates as a function of cue set size, display set size, and target-distractor congruency**.

**Display set size**	**Cue set size**			
	**2-cue**		**6-cue**	
	***C***	***I***	***C***	***I***
**REACTION TIMES (ms)**				
2-letter	595 (26)	617 (25)	651 (31)	747 (35)
6-letter	600 (25)	640 (26)	789 (41)	839 (38)
**ERROR RATES (% INCORRECT)**				
2-letter	4.4 (0.9)	6.6 (1.2)	5.2 (0.8)	9.1 (1.4)
6-letter	5.2 (1.0)	6.7 (1.1)	13.9 (1.6)	17.9 (1.8)

C, Congruent; I, Incongruent. Standard errors are in the parentheses.

To clarify the Three-Way interaction, we conducted two separate ANOVAs, one for the data in the 2-cue condition and the other for the data in the 6-cue condition. In the 6-cue condition, all the effects were significant. RT was longer in the 6-letter condition (814 ms) than in the 2-letter condition (699 ms), *F*_(1, 19)_ = 80.65, *MS*_*e*_ = 3238, *p* < 0.001, η^2^_*p*_ = 0.81, and on incongruent (793 ms) than congruent (720 ms) trials, *F*_(1, 19)_ = 53.88, *MS*_*e*_ = 1988, *p* < 0.001, η^2^_*p*_ = 0.74. DisplaySize interacted with Congruency, *F*_(1, 19)_ = 7.58, *MS*_*e*_ = 1404, *p* < 0.02, η^2^_*p*_ = 0.29. The congruency effect was larger in the 2-letter condition (96 ms) than in the 6-letter condition (50 ms), indicating a significant dilution or perceptual load effect.

The pattern of data differed in the 2-cue condition. The main effects of DisplaySize and Congruency were both significant, with faster RT on the 2-letter trials (606 ms) than on the 6-letter trials (620 ms), *F*_(1, 19)_ = 16.21, *MS*_*e*_ = 247, *p* < 0.001, η^2^_*p*_ = 0.46, and on the congruent trials (598 ms) than on the incongruent trials (629 ms), *F*_(1, 19)_ = 20.11, *MS*_*e*_ = 960, *p* < 0.001, η^2^_*p*_ = 0.51. The interaction between DisplaySize and Congruency was marginally significant, *F*_(1, 19)_ = 4.04, *MS*_*e*_ = 360, *p* = 0.06, η^2^_*p*_ = 0.18. Importantly, the direction of the interaction was opposite to what was found in Experiment 1: the congruency effect was larger in the 6-letter condition (40 ms) than in the 2-letter condition (22 ms). Thus, there was no evidence of a dilution effect when the neutral letters were outside the attentional focus in the 2-cue condition.

To confirm statistically that the pattern of data in Experiment 1 differed from that in Experiment 2, we conducted a combined analysis of the RT data across the two experiments, using a mixed ANOVA with Experiment as a between-subjects factor and CueSize, DisplaySize, and Congruency as within-subjects factors. For the sake of brevity, we report only the significant interactions with Experiment, of which there were two. One was a significant interaction between DisplaySize and Experiment, *F*_(1, 36)_ = 9.38, *MS*_*e*_ = 3582, *p* < 0.01, η^2^_*p*_ = 0.21, suggesting that the increase in RT from the 2-letter to 6-letter condition was larger in Experiment 1 (an increase of 106 ms) than in Experiment 2 (an increase of 65 ms). The second was a significant four-way interaction, *F*_(1, 36)_ = 4.49, *MS*_*e*_ = 946, *p* < 0.05, η^2^_*p*_ = 0.11. Subsequent analyses on the 2-cue and 6-cue trials separately indicated that the 4-way interaction arose primarily from the participants in the two experiments behaving differently in the 2-cue condition, where a significant 3-way interaction of DisplaySize, Congruency, and Experiment was found, *F*_(1, 36)_ = 5.56, *MS*_*e*_ = 473, *p* < 0.05, η^2^_*p*_ = 0.13. A similar 3-way interaction was not found in the 6-cue condition, *F*_(1, 36)_ = 1.59, *MS*_*e*_ = 1042, *p* = 0.21, η^2^_*p*_ = 0.04. These results confirmed that the pattern of data in Experiments 1 and 2 differed when the cue set size was 2, but not when it was 6.

### Discussion

The results of Experiment 2 suggest that the extent of attentional focus modulates the effect of display set size on distractor processing. In the 6-cue condition, the target was equally likely to appear at any location in the search array. To find the target quickly, the best strategy would be to adopt a relatively broad attentional focus that would include the entire target display, including the neutral stimuli. As the neutral stimuli were within the attentional focus, they would compete with the critical distractor for representation. Hence, a dilution effect was found in the 6-cue condition. In contrast, in the 2-cue condition, the participants' attention was likely to be more narrowly focused, and unlike Experiment 1, there was no abrupt luminance increment to draw attention more widely when the target array appeared. As the letters that appeared at the uncued locations were largely outside the focus of attention, they would not receive the same kind of processing as their counterparts in the 6-cue condition. Whatever processing these letters might have received due to attentional leakage, the level of processing was not sufficient to interfere with the representation of the distractor. As a result, increasing display set size in the 2-cue condition did not lead to a dilution effect.

It is worth noting that the participants in the 2-cue condition of Experiment 2 took longer to respond to the target on the 6-letter trials than on the 2-letter trials despite the fact that the participants knew in advance that the target would never occur at an uncued location. The increased RT in the 6-letter trials indicated that attention could not completely filter out all the irrelevant information. This result is in line with the view that attentional selection is often incomplete, and that some processing can still happen to irrelevant stimuli even with clear spatial separation between a target and irrelevant distractors (Treisman, 1964; Miller, 1991).

Another interesting aspect of Experiment 2's data is the reversed dilution effect in the 2-cue condition. The congruency effect was larger, instead of smaller, when the display consisted of 6 letters rather than 2 letters. It is notable that RT was substantially longer on the 6-letter trials compared with the 2-letter trials. As we discussed in Experiment 1, an increase in response latencies increases the window of opportunity for distractor intrusion. As a result, congruency effect was larger in the 6-letter condition than in the 2-letter condition.

## Experiment 3

As mentioned earlier, several researchers have proposed a dilution account to interpret the reduction in distractor interference with increasing display set size (Benoni and Tsal, 2010; Tsal and Benoni, 2010; Wilson et al., 2011). Because stimuli of the same category, which share both basic features and response code, were used in these prior studies, the proposed dilution accounts emphasize competition between the features of the added display items and the features of the distractor, which degrades the quality of the distractor representation (e.g., Tsal and Benoni, 2010). In other words, they suggest that dilution occurs at a feature level.

Experiment 3 was designed to test this notion empirically. In Experiment 3, both the 2-item and 6-item conditions had 2 upright letters present in the target array, but in the 6-item condition, there were also 4 inverted letters. Because the inverted letters shared basic features but not meaning with the critical distractor, this design allowed us to assess the effect of neutral stimuli on distractor processing at a feature level. If dilution occurs at a feature level, the participants in Experiment 3 should show the same pattern of result as that in Experiment 2. Conversely, if dilution occurs at a level beyond feature processing (e.g., at a categorical, semantic, or response level), no dilution effects should be found in the 6-item condition.

### Method

The method of Experiment 3 was identical to that of Experiment 2 except for the stimuli in the large display set size condition. Instead of 6 letters, the search array consisted of 2 upright letters (i.e., the target and a neutral letter selected randomly on each trial from the set of neutral stimuli as in Experiment 2) and 4 inverted letters constructed from the original set of neutral letters (i.e., P, F, U, L, E) with a 180 degree rotation. As before, we varied the cue set size (the 2-cue and 6-cue conditions) independently of the display set size (the 2-item and 6-item conditions). Sixteen new participants volunteered for the experiment.

### Results

**Figure 5:**
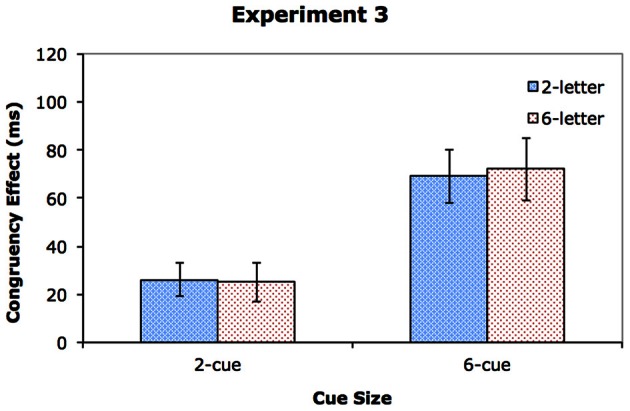
**The congruency effect for Experiment 3**.

Table 5 shows the response times and error rates, and Figure 5 shows the congruency effects. As before, we conducted two separate repeated-measures ANOVAs, one on the RT data (see Table 2), and the other on the error rates (see Table 3). The participants were again faster and more accurate in the 2-cue condition (615 ms with 4.8% error rate) than in the 6-cue condition (761 ms with 11.5% error rate), *p* < 0.001 for both RT and accuracy. They were also faster and more accurate when the display set size was 2 (660 ms with 6.0% error rate) rather than 6 (715 ms with 10.2% error rate), *p* < 0.001 in both cases. In addition, responses were faster and more accurate on congruent trials (664 ms with 7.1% error rate) than on incongruent trials (712 ms with 9.1% error rate), *p* < 0.001 for RT, and *p* < 0.02 for accuracy. The interaction between CueSize and DisplaySize was also significant, *p* < 0.001 for RT and accuracy. This suggests that once again, an increase in display set size impaired performance more in the 6-cue condition (an increase of 105 ms and 8% error rate) compared with the 2-cue condition (an increase of only 6 ms and 0.4% error rate). CueSize interacted with Congruency in RT, *p* < 0.001, indicating a larger congruency effect in the 6-cue condition (71 ms) than in the 2-cue condition (26 ms). Importantly, neither the two-way interaction between DisplaySize and Congruency nor the 3-way interaction of CueSize, DisplaySize and Congruency was significant, *F*_(1, 15)_ < 1, *ns*. in both cases. These results indicate that the presence of the inverted letters had a negligible effect on the degree of distractor interference regardless of whether the cue set size was 2 or 6. No other effects reached significance, and there was no evidence of a speed-accuracy tradeoff.

**Table 5:** **Experiment 3: mean reaction times and error rates as a function of cue set size, display set size, and target-distractor congruency**.

**Display set size**	**Cue set size**			
	**2-cue**		**6-cue**	
	***C***	***I***	***C***	***I***
**REACTION TIMES (ms)**				
2-item	599 (29)	625 (31)	674 (35)	743 (34)
6-item	605 (31)	630 (33)	777 (33)	849 (38)
**ERROR RATES (% INCORRECT)**				
2-item	4.1 (0.7)	5.0 (1.0)	5.8 (1.0)	9.1 (1.5)
6-item	4.4 (0.9)	5.5 (1.5)	14.0 (1.6)	16.9 (2.4)

Standard errors are in the parentheses. C, Congruent; I, Incongruent.

A combined analysis across Experiments 2 and 3 was conducted on the RT data to verify that the pattern of data in the two experiments differed significantly. Again, for the sake of brevity, we report only the significant interactions that involve Experiment. The only significant effect was the four-way interaction of CueSize, DisplaySize, Congruency, and Experiment, *F*_(1, 34)_ = 6.12, *MS*_*e*_ = 819, *p* < 0.05, η^2^_*p*_ = 0.15. Separate analyses on the 2-cue and 6-cue trials confirmed that the four-way interaction in the original analysis arose from the 6-cue condition, where a significant three-way interaction of DisplaySize × Congruency × Experiment was found, *F*_(1, 34)_ = 4.95, *MS*_*e*_ = 1086, *p* < 0.05, η^2^_*p*_ = 0.13. A similar effect was not found in the 2-cue condition, *F*_(1, 34)_ = 2.0, *MS*_*e*_ = 356, *p* = 0.17, η^2^_*p*_ = 0.06. These results suggest that the effect of neutral stimuli on distractor processing differed in the 6-cue condition between Experiments 2 and 3.

### Discussion

The most important finding of Experiment 3 was the elimination of the dilution effect in the 6-cue condition. Adding inverted letters to the display did not lower the distractor interference in this condition, even though the upright letters added to displays in the same condition of Experiments 1 and 2 lowered the distractor interference in those experiments. This result suggests that the inverted letters in the 6-item condition had a negligible effect on the degree of distractor processing, despite the fact that they increased the overall RT to the target. This RT increase likely reflects the extra difficulty in locating the target due to the increased similarity between the target and the relevant items in the search array. Previous research has shown that an increase in similarity between a target and distractors impairs segmentation, making it hard to distinguish the target from the distractors (Duncan and Humphreys, 1989, 1992). Thus, these items that have been added to the display, which share features with the target and the critical distractor but do not activate responses in the same category, can delay the response to the target but do not necessarily degrade the representation of the distractor.

The absence of a dilution effect in Experiment 3 also suggests that the locus of dilution in Experiments 1 and 2 probably occurred at a semantic level. That said, caution must be taken in generalizing this result to other experimental paradigms. It is quite possible that the locus of dilution depends on participants' behavioral goals. When a task requires a categorical or semantic level of processing, dilution may occur at these levels. However, when a task requires a feature level of processing, dilution may occur at the feature level. In the present study, although the two target letters could be distinguished on the basis of basic features, they were referred to as individual letters H and S. Naming the letters would likely induce the participants to code them at a semantic level, differentiating them from the inverted letters in terms of task relevancy and avoiding dilution from the inverted letters in the 6-cue condition.

## Experiment 4

Experiment 3 showed that dilution effects could be eliminated when neutral stimuli did not share the same response code as the target and distractor. In Experiment 4, we investigated whether dilution effects could also be eliminated when participants had preknowledge of the target color. We reasoned that knowing the color of the target in advance would enable participants to use that information to direct their attention to those stimuli that had the task relevant color, thereby excluding the stimuli that had the task irrelevant color from the attention focus. Consequently, if the additional neutral letters in the 6-letter display had a task irrelevant color, they should not affect the degree of distractor processing even when all the locations in the search array were cued in the 6-cue condition. To test this hypothesis, the participants were divided into two groups in Experiment 4. One group (the predictable group) knew in advance the color of the target on each trial. The target was red in one block, and green in a different block. The other group (the unpredictable group) had no preknowledge of the target color on a given trial. The target was equally likely to be red or green. On all trials, 6 locations were cued. If a dilution effect was found in the unpredictable group but not in the predictable group, this would provide additional evidence that the extent of attentional focus modulates dilution effects.

### Method

The method was similar to that of Experiment 2 except for the following differences. First, as dilution was found only when participants adopted a relatively wide attentional focus, Experiment 4 included only the 6-cue condition. Second, the stimuli in the target display were either red (RGB = 255, 64, 64) or green (RGB = 64, 255, 64). In all conditions, the target had the same color as only one other stimulus: the neutral letter at its opposite location. In other words, the target display consisted of either 2 red and 5 green stimuli, or 2 green and 5 red stimuli. Finally, the participants were randomly and equally divided into two groups. For the predictable group, the color of the target was the same throughout the trials within a block. Half of them completed the red block before the green one, and the order of the blocks was reversed for the other half. For the unpredictable group, the color of the target was unknown on a given trial. The target was equally likely to be red or green within a block. Twenty new participants took part in the experiment.

### Results

**Figure 6:**
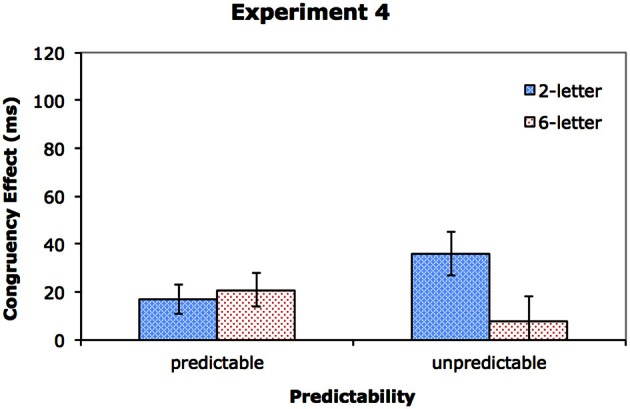
**The congruency effect for Experiment 4**.

The data from one participant in the predictable group was excluded from analyses due to high error rates (averaged over 20% across all conditions). Table 6 shows the response times and error rates, and Figure 6 shows the congruency effects. A mixed ANOVA with DisplaySize and Congruency as within-subjects factors and Group as a between-subjects factor was performed on the RT data (see Table 7). The results show that RT was faster in the predictable group (581 ms) than in the unpredictable group (701 ms), *p* < 0.05, indicating that knowing the target color in advance facilitated target responses. As in previous experiments, RT was faster in the 2-letter condition (630 ms) than in the 6-letter condition (651 ms), *p* < 0.001, and in the congruent condition (630 ms) than in the incongruent condition (651 ms), *p* < 0.01. DisplaySize interacted with Group, *p* < 0.01, suggesting a larger set size effect for the unpredictable group (an increase of 33 ms) than for the predictable group (an increase of 8 ms). In addition, there was a significant three-way interaction of DisplaySize, Congruency, and Group, *p* < 0.05, which is illustrated in Figure 6.

**Table 6:** **Experiment 4: mean reaction times and error rates as a function of the preknowledge of the target color, display set size, and target-distractor congruency**.

**Display set size**	**Target color**			
	**Predictable**		**Unpredictable**	
	***C***	***I***	***C***	***I***
**REACTION TIMES (ms)**				
2-letter	568 (27)	585 (26)	666 (32)	702 (32)
6-letter	574 (29)	595 (25)	713 (38)	721 (32)
**ERROR RATES (% INCORRECT)**				
2-letter	3.3 (1.2)	2.9 (0.6)	7.1 (1.1)	6.8 (1.4)
6-letter	3.7 (0.7)	4.1 (0.7)	8.5 (1.2)	10.0 (1.4)

Standard errors are in the parentheses. C, Congruent; I, Incongruent.

**Table 7:** **Results of statistical analysis of the reaction times and error rates in Experiment 4**.

	**Reaction times**			**Error rates**		
	***F*_(1, 17)_**	***p***	**η^2^_*p*_**	***F*_(1, 17)_**	***p***	**η^2^_*p*_**
Group	7.83^*^	0.02	0.32	12.30^**^	0.01	0.42
Display	22.72^***^	0.001	0.57	8.02^*^	0.02	0.32
Display^*^Group	9.17^**^	0.01	0.35	1.80	0.20	0.10
Cong	15.42^***^	0.001	0.48	0.34	0.57	0.02
Cong^*^Group	0.09	0.77	0.01	0.46	0.51	0.03
Display^*^Cong	4.26	0.06	0.20	1.93	0.18	0.10
Display^*^Cong^*^Group	7.86^*^	0.02	0.32	0.32	0.58	0.02

Display, DisplaySize; Cong, Congruency.

* p < 0.05;

** p < 0.01;

*** p < 0.001.

To clarify the three-way interaction, two separate ANOVAs, one for each group, were performed. For the predictable group, while the main effect of congruency was significant, *F*_(1, 8)_ = 10.68, *MS*_*e*_ = 298, *p* < 0.05, η^2^_*p*_ = 0.57, neither the effect of DisplaySize nor its interaction with Congruency reached significant, *p* > 0.1 in both cases. The predictable group thus showed no evidence of a dilution effect. For the unpredictable group, in addition to the main effects of congruency, *F*_(1, 9)_ = 6.88, *MS*_*e*_ = 696, *p* < 0.05, η^2^_*p*_ = 0.43, and DisplaySize, *F*_(1, 9)_ = 22.21, *MS*_*e*_ = 508, *p* < 0.01, η^2^_*p*_ = 0.71, there was a significant interaction between the two factors, *F*_(1, 9)_ = 10.73, *MS*_*e*_ = 175, *p* < 0.01, η^2^_*p*_ = 0.54, with a larger congruency effect in the 2-letter condition (36 ms) than in the 6-letter condition (8 ms), suggesting dilution.

A mixed ANOVA was also performed on the error rates (see Table 7). Consistent with the RT results, responses were more accurate in the predictable group (3.5% error rate) than in the unpredictable group (8.1% error rate), *p* < 0.01, and on the 2-letter trials (5% error rate) than on the 6-letter trials (6.6% error rate), *p* < 0.05. No other results were significant.

### Discussion

The most important finding of Experiment 4 was that preknowledge of the target color could eliminate dilution effects. Whereas a dilution effect was found when participants had no advanced knowledge of the target color, the effect was negligible when the target color was predictable on a given trial. These results are consistent with the notion that attentional focus modulates the effect of display set size on distractor processing. When the color of the target was known in advance, the participants could use this knowledge to deploy attention efficiently. Thus, even though the attentional focus induced by the cue was wide enough to include all the stimuli, the preknowledge of the target color would allow the participants to locate the task relevant color quickly and to adjust their attentional focus accordingly. This means that the neutral letters with the task irrelevant color could be excluded from the attentional focus fairly early in the process, thereby minimizing their effect on distractor processing.

In contrast, the participants in the unpredictable group did not know the target color in advance. For them to use color to guide attention, they would have to first ascertain the task relevant color by determining which color was the minority color and which one was the majority color, which would probably take some time. As attention could not be zoomed in to the target quickly, the irrelevant letters had more opportunity to be processed, resulting in the dilution effect in the unpredictable group.

It is worth noting that although the distractor differed from the target in both color and location, this perceptual segregation did not completely shield the distractor from being processed, as evidenced by the significant congruency effect in both the predictable and unpredictable groups. This distractor interference suggests that the attentional focus included the distractor along with the two cued locations on either side of it^2^. A similar result was reported by Harms and Bundesen (1983), who found a significant response congruency effect despite the fact that the target and distractors differed in both color and spatial locations.

Tsal and Benoni (2010, Experiment 3) have also investigated the effect of preknowledge of the target color on distractor processing. In two of their experimental conditions most relevant to the present experiment, i.e., the high load and dilution conditions, Tsal and Benoni's participants saw multi-stimulus displays that consisted of letters of different colors. Whereas the color of the target was unknown on a given trial in the high load condition, it was known in advance in the dilution condition. Although the average RT was substantially slower in the high load condition than in the dilution condition, no congruency effect was found in either condition. In contrast, a significant congruency effect was found in the low load condition, in which the target display consisted of a single colored target letter and one distractor. Similar results were found by Benoni and Tsal (2010, Experiment 2). Once again, no significant congruency effects were found in either the high load or dilution condition, but only in the two low load conditions. (The two conditions differed in that the color of the target was known in one condition but not in the other condition). These results confirmed the researchers' hypothesis that perceptual load did not influence the degree of distractor processing when the number of neutral items was held constant. Based on their results, Benoni and Tsal also concluded that whereas preknowledge of target location affects both target and distractor processing, preknowledge of target color affects only target processing.

In Experiment 4 of the present study, the pattern of data between the predictable and unpredictable groups differed not only in the overall response latencies to the target (longer in the unpredictable than the predictable group), but also in the effect of display set size on distractor processing. Whereas the magnitude of the congruency effect decreased with an increase in display set size in the unpredictable group, there was no evidence that display set size influenced the degree of distractor processing in the predictable group. These results suggest that preknowledge of the target color affected both target and distractor processing in our paradigm. However, because of the many differences in methodology between the present experiment and the experiments of Benoni and Tsal (2010) and Tsal and Benoni (2010), we do not consider our results contradictory to their claim. Our results simply show that under some conditions, preknowledge of target color can affect participants' deployment of attention, which in turn can influence the degree of distractor processing.

## General discussion

These experiments, and the earlier experiments that they build on, illustrate the complexity of visual processing in multi-element displays with targets and distractors. Attention can select the targets once they are identified, but in many cases it cannot prevent the distractors from being partially processed and interfering with the target. This small bit of processing accorded to the distractor is not guaranteed, however; it can be blocked if extra items are added to the display. The current experiments show that these extra items are themselves subject to changes in attentional allocation triggered by sudden luminance increment and by expectations about target color.

Sorting out these different effects will require an understanding of the different factors governing distractor processing in complex displays. One key question in recent years has been why the interference from an irrelevant distractor diminishes when more items are added to the display. The original perceptual load theory posited that these extra items required processing as part of the task, which took processing resources away from the distractor. However, experiments by Tsal and Benoni (2010) and by Wilson et al. (2011) showed that the extra items can weaken distractor interference even when they are easily identified as irrelevant to the task. They described the effect as dilution, because the mere presence of these items diluted the interfering effects of the distractor, independent of their relevance to the task.

Wilson et al. (2011) explained dilution within a two-stage account in the style of Neisser (1967) and Hoffman (1979), with the dilution occurring in the second stage, after the target has been identified and selected. In this account, any processing resources not used by the target are allocated to the non-target items, but unlike Lavie's account, these non-target items are all equal in that their original relevance to the task does not affect their processing. The more non-target items there are, the more interference each item encounters.

Wilson et al.'s (2011) account predicts that Experiment 2 should show dilution in the 2-cue condition; when extra items are present in the 6-letter display, they should decrease the distractor interference relative to the 2-letter display. Instead, the luminance-decrement items in Experiment 2 do not dilute the effects of the distractor, demonstrating that dilution in this paradigm depends on the attentional effects of the display onsets. All items do not contribute equally to dilution; it depends on whether they benefit from the attentional focus or not. These results are consistent with prior research showing that inducing participants to adopt a small attentional focus so that distractors fall outside it could minimize distractor interference in a search display (e.g., Eriksen and St. James, 1986; Yantis and Johnston, 1990; LaBerge et al., 1991). They are also consistent with the more recent finding that singletons capture attention when they are inside but not outside the attentional focus (Belopolsky et al., 2007; Belopolsky and Theeuwes, 2010).

The effects of attention are also seen in Experiment 4, in which dilution from the non-targets is eliminated if their color makes it easy to ignore them. The two types of attentional effects on dilution shown in these experiments are consistent with Yeh and Lin's (2013) demonstration that dilution is affected by perceptual grouping. One option for explaining both sets of results is to modify the dilution account to allow for attentional effects on all elements in the display at some stage of processing. In other words, the amount of dilution from a particular display item will depend on its location relative to the attentional focus, its grouping with other elements in the display, its features that match the expected features of the target, and other factors that affect attentional allocation. Another option to account for these data is to modify the perceptual load account to include a detailed description of how the different non-targets in the display interact to affect one another's processing. As Yeh and Lin have suggested, it may be possible to construct an account somewhere in between the pure perceptual load theory and the pure dilution theory that can explain all of these different experimental results, but it is likely to include a combination of factors that make it more complex than either of those original theories.

While Experiments 2 and 4 show that dilution is affected by the attentional focus, Experiment 3 demonstrates another informative aspect about dilution: that it occurs not because the basic features of the non-targets interfere with processing the features of the distractor, but because the non-target are activating letter representations that compete with the representation for the distractor letter. When the non-targets are inverted so that they do not match any letter representation, the competition is eliminated. The interference that underlies these effects appears to arise at the level of letter representations, and not lower down at the level of simple features.

These results give us a clearer view of how dilution occurs in the processing of multi-element displays, and how it can be prevented. As shown by Tsal and Benoni (2010) and by Wilson et al. (2011), items can contribute to dilution even when their location makes it clear that they are irrelevant to the task, but only if a sudden increment in luminance draws a certain amount of attention to them. Furthermore, the effects of a letter will only be diluted by other letters in the display, and not by items sharing basic features with the letters. Thus, dilution is not as widespread or as uniform as previous accounts predict. These results, like those of Yeh and Lin (2013), suggest that within multi-element displays, there is a complex interaction between the separate elements as they all compete for some level of attention, and that the allocation of attention is shaped by multiple factors working simultaneously.

### Conflict of interest statement

The authors declare that the research was conducted in the absence of any commercial or financial relationships that could be construed as a potential conflict of interest.

## Footnotes

^1^In all experiments, response latencies greater than 2000 ms were excluded. These constituted less than 1% of the total data in each experiment. Only trials that were correct were included in the tables and the statistical analyses.

^2^Although our study was not designed to test the issue of attentional selection over contiguous vs. non-contiguous regions, there is evidence that either type of selection may occur under the right circumstances (see Jans et al., 2010; and Cave et al., 2010, for a review). The fact that we found substantial congruency effects in all our experiments despite the positional certainty of the distractor indicates that attention selected contiguous regions in the present experimental paradigm.
